# Dual contraceptive use and associated factors among female sex workers in Gulu City, Uganda in 2023

**DOI:** 10.1186/s41182-023-00536-6

**Published:** 2023-08-16

**Authors:** Felix Bongomin, Pebalo Francis Pebolo, Winnie Kibone, Pamela Okwir Apio, Lauryn Nsenga, Jerom Okot, Grace Madraa, Fiona Gladys Laker, Martha Akello, Simple Ouma, David Musoke

**Affiliations:** 1 Department of Medical Microbiology and Immunology, Faculty of Medicine, Gulu University, P.O. Box 166, Gulu, Uganda; 2 Department of Reproductive Health, Faculty of Medicine, Gulu University, Gulu, Uganda; 3 School of Medicine, College of Health Sciences, Makerere University, Kampala, Uganda; 4 Makerere Lung Institute, School of Medicine, Makerere University, Kampala, Uganda; 5 School of Medicine, Kabale University, Kabale, Uganda; 6 Department of Rural Development and Agri-Business, Faculty of Agriculture, Gulu University, Gulu, Uganda; 7 Urban Research and Consultancy East Africa Limited, Kampala, Uganda; 8 Voice of Community Empowerment, Gulu, Uganda; 9 The AIDS Support Organization, Kampala, Uganda; 10 The Institute of Clinical Trials and Methodology, University College London, London, UK; 11 Department of Pharmacology and Therapeutics, Faculty of Medicine, Gulu University, P.O. Box 166, Gulu, Uganda

**Keywords:** Female sex workers, Dual contraceptive, Condoms, Implants

## Abstract

**Background:**

Dual contraception is an essential approach to mitigating the heightened risks of unintended pregnancies and sexually transmitted infections faced by female sex workers (FSWs). We determined the prevalence and factors associated with dual contraceptive use among FSWs in Northern Uganda.

**Methods:**

A cross-sectional study was conducted in Gulu city, Uganda between February, and March 2023. Purposively selected FSWs aged 18 years or older who provided verbal informed consent were enrolled in the study. A sample size of 374 was estimated. Data on sociodemographic and reproductive health characteristics were collected using an interviewer-administered semi-structured questionnaire. Current dual contraception was defined the as concurrent use of a barrier and any other modern contraceptive methods within the last 3 months. Continuous variables were summarized using mean and standard deviation and categorical variables were summarized using frequencies and percentages. Modified Poisson regression analysis was used to determine factors associated with dual contraceptive use.

**Results:**

We enrolled 314 FSWs, with a mean age of 28.8 ± 6.4 years. About 13.8% (*n* = 41) were living with HIV. In total, 66.2% (95%CI 60.8–71.3) of the participants (*n* = 208) reported current dual contraceptive use. The most common modern contraceptive method used was the implants (52.4%, *n* = 109), followed by injectable contraceptives (22.6.0%, *n* = 47), and bilateral tubal ligation (0.5%, *n* = 1) was the least utilized. Having had at least one previous pregnancy was positively associated with dual contraceptive use (adjusted PR: 1.87, 95%CI 1.40–2.51, *p* < 0.001).

**Conclusions:**

A high proportion of FSWs in Gulu city were currently using dual contraceptives. Interventions are needed to facilitate the use of dual contraception, particularly among FSWs without a history of previous pregnancies.

## Introduction

Dual contraception (use of condom plus another modern contraceptive method) is an effective means of preventing sexually transmitted infections (STIs) including HIV and unintended pregnancies [1]. However, an earlier study among female sex workers (FSWs) in Uganda showed that only 45% of FSWs in Gulu district used dual contraception [2]. This prevalence is below the national estimate of 58% utilization of dual contraceptives among FSWs in Uganda [3]. From literature, several factors have been implicated in the low utilization of dual contraceptives among FSWs including myths and misconceptions associated with contraceptives, alcohol and substance abuse, low level or education, exploitation and, stigma and discrimination among FSWs [4–6]. In addition, rush of sexual negotiations with clients [7], having a non-emotional partner, lack of social support, rate, substance abuse, and young age [8] also contribute to low use of dual contraception among FSWs. If unattended to, the low use of dual contraception [2, 9] puts FSWs at greater risk of STIs, unwanted pregnancies [3, 10], induced abortion and its complications [11].

In sub-Saharan Africa, the risk of HIV infection is 12-folds higher among FSWs than it is in the general population [12]. In Uganda, the prevalence of HIV among sex workers is currently estimated at about 31.3% compared to the 6.3% in the general population [13]. In the bid to achieve the sustainable development goal (SDG) indicator 3.7.1 on contraceptive use, the Uganda Ministry of Health and development partners have greatly increased the availability of free modern contraceptives. Thus, more resources have been allocated to increase access to condoms for HIV prevention despite its high contraceptive failure rates (17%) compared to other modern conceptive methods [14].

Joint efforts from The AIDS Support Organization (TASO) and Reproductive Health Uganda (RHU) have significantly improved access to sexual and reproductive health and rights services among FSWs in Gulu City. However, despite integrating HIV and Sexual and reproductive health services for FSWs across the country, more effort to enhance the use of dual contraception among FSWs is needed as current use is still below average [3]. This study, therefore, aimed to determine the prevalence and associated factors with dual contraceptive use among FSWs of reproductive age in a fast-growing urban city in Northern Uganda.

## Methods

### Study area

Gulu city is a fast growing, mainly urban city in Northern Uganda, with an estimated population of 150,000 inhabitants. It is located about 350 kms from Kampala, the capital city of Uganda. The study was conducted in several hotspots within Gulu, city which has an estimated 1300 FSWs. A total of 15 hotspots across the city constituted the study sites. Participants were recruited from different locations within Gulu City, where FSWs were known to congregate, including bars, nightclubs, brothels, and streets in pece, layibi and laroo divisions. A vast majority of the FSWs in Gulu and surrounding districts live and work in Gulu city [15]. There are over 20 governmental and non-governmental health facilities and organizations that work closely with FSWs in Gulu city, including Gulu Regional Referral Hospital, The AIDS Support Organization (TASO)—Gulu Branch, Reproductive Health Uganda, the Voice of Community Empowerment, and others.

### Study design and period

Between February, and March 2023, we conducted a community-based cross-sectional study among eligible FSWs in Gulu city, Northern Uganda. This report is written in accordance with the Strengthening the Reporting of Observational Studies in Epidemiology (STROBE) guidelines for cross-sectional studies [16].

### Population

The source population were FSWs of reproductive age engaged in sex work within Gulu city. To be eligible for the study, participants had to be 18 years or older and within the reproductive age bracket (15–49 years), cisgender female, provided written informed consent to participate in the study and currently working and identifying as a sex worker. HIV status was verified from the most recent HIV testing results less than 3 months old. We excluded transgender women.

### Sample size estimation

The sample size of 374 was calculated using the modified Kish-Leslie formula (1965) for a single population, with the prevalence of dual contraception estimated at 58% from a previous study [8], type 1 error of 5% (1.96) and 95% confidence. Consecutive sampling was used to recruit the participants in the study.

### Data collection tool and procedures

Enrolment was conducted by engaging peer leaders of a hotspot who knew most of the FSWs who operate within his/her area. Two research assistants (MA and FGL) had previous and ongoing engagements with FSWs in Gulu through the Voice of Community Empowerment, which provides advocacy for FSWs and other vulnerable populations in Gulu. Additional respondents were enrolled through snowballing, where after interviewing the participants, they were requested to identify and refer their colleagues to the research team.

Data were collected through face-to-face interviews in a private space within the place of sex work across the hotspots using a pre-tested semi-structured questionnaire. The questionnaire was developed in English and translated into the local language, *Acholi*. The questionnaire collected information on socio-demographic characteristics, sexual behaviour, condom use, contraceptive use, and depression. The data collection process was supervised by FB and GM.

### Operational definition

1. Dual contraceptive user was defined as a FSW who concurrently use a barrier (male or female condoms) regularly and any other modern (pills, injections, intrauterine devices) contraceptive methods.
2. Depression over the past 2 weeks was assessed using the Patient Health Questionnaire-2 (PHQ-2), with a cutoff score of 3 used to define depressed mood [17].
3. Condom use was defined as regularly if a FSW uses it most of the time or “sometimes” if the use was occasional and “never” if the participants never used it in the past 3 months.

### Statistical analysis

Data were cleaned and exported into STATA version 14.0, StataCorp LLC, College Station, Texas, USA for analysis. Normally distributed continuous variables were summarized using mean and standard deviation and categorical variables were summarized using proportions and percentages. The proportion FSWs with dual contraceptive use was summarized as a proportion with its 95% confidence interval. For the regression analysis, the outcome variable of dual contraceptive use was dichotomized as 1 “yes” 0 “no”. Since the outcome was common (the proportion of dual contraceptive use was > 10%), modified Poisson regression was used to determine factors associated with dual contraception and measure of association was the prevalence ratio. We conducted bivariate analysis and variables with *p* < 0.20 were considered for multivariate analysis. In the multivariate analysis, we used manual backward elimination method until all the variables in the model had *p* value ≤ 0.05. We assessed for interaction by forming product terms and performed a chunk test. We assessed for confounding by considering a percentage change of > 10% in the crude and adjusted prevalence ratios. The goodness of fit of the model was assessed using Hosmer–Lemeshow goodness of fit test. Variables with *p* values < 0.05 were considered statistically significant.

### Ethical consideration

The study received ethical approval from the Gulu University Research Ethics Committee (approval number: GUREC-2022-414). Verbal informed consent was obtained from all participants before the start of the study. Participants were informed of their right to withdraw from the study at any time and that their participation was voluntary. All data collected was kept confidential and anonymous. The ethical principles outlined in the *Declaration of Helsinki* were all adhered to. Study participants who required specific sexual and reproductive health services were counselled and referred to the appropriate health facility.

## Results

### Participants

Of the 374 anticipated participants, 314 (response rate 84%) were eligible and were enrolled into the study.

### Sociodemographic characteristics

Overall, 314 FSWs with a mean age of 28.8 ± 6.4 years were included in the final analysis. More than half of the participants were below the age of 29 (58.3%, *n* = 183). In terms of education, slightly over half of the participants had received secondary or tertiary education (51.3%, *n* = 161). Most participants resided in rural areas (93.3%, *n* = 293), were single (81.8%), and Christians (92.6%), while only a small proportion identified as Muslim (7.4%). About two-third (67.2%, *n* = 211) had been pregnant between 1 and 4 times, and 9.5% had been pregnant more than four times. In terms of abortion, 39.2% (*n* = 123) had had between 1 and 4 abortions.

When it comes to income, 40.5% (*n* = 127) of participants reported sex work as their major source of income. In terms of mental health, most participants reported symptoms of depression (90.1%, *n* = 283). In terms of awareness of HIV status, most participants were aware of their status (94.9%, *n* = 298). Of those who were aware, 13.8% (*n* = 41) were living with HIV. Regarding alcohol consumption, 80.7% (*n* = 250) of participants reported consuming alcohol sometimes, while 16.8% (*n* = 52) reported consuming alcohol always. In terms of age at first sexual encounter, slightly over half of the participants had their first sexual encounter before the age of 17 (55.2%, *n* = 158). Table 1 summarizes the baseline characteristics of the study participants.

**Table 1:** Sociodemographic and reproductive health characteristics of female sex workers in Gulu city, Uganda

Variable	Category	Frequency (*N* = 314)	Percent (%)
Age (years) mean (SD)		28.8 (6.4)	
Age (years)	< 29	183	58.3
	> 28	131	41.7
Education	None/Primary	153	48.7
	Secondary/tertiary	161	51.3
Residence	Urban	21	6.7
	Rural	293	93.3
Marital status	Married	57	18.2
	Single	257	81.8
Religion	Moslem	23	7.4
	Christian	286	92.6
Number of pregnancies	None	73	23.3
	1–4	211	67.2
	> 4	30	9.5
Number of abortions	None	167	53.2
	1–4	123	39.2
	> 4	24	7.6
Number of unplanned pregnancy *n* = 305	None	166	54.4
	1–3	118	38.7
	> 3	21	6.9
Number of conceptions during sex work *n* = 305	None	193	63.3
	1–3	100	32.8
	> 3	12	3.9
Number of unplanned pregnancies during sex work *n* = 307	None	187	60.9
	1–4	108	35.2
	> 4	12	3.9
Sex work as major source of income	No	187	59.6
	Yes	127	40.5
Depression	No	31	9.9
	Yes	283	90.1
Awareness of HIV status	No	16	5.1
	Yes	298	94.9
HIV status *n* = 298	Negative	257	86.2
	Positive	41	13.8
Alcohol consumption *n* = 310	Always	52	16.8
	Never	8	2.6
	Sometimes	250	80.7
Age at first sexual encounter *n* = 286	< 17	158	55.2
	> 16	128	44.8

### Reproductive characteristics

Regarding unplanned pregnancy and sex work, 38.7% (*n* = 118) had experienced between 1 and 3 unplanned pregnancies, and 6.9% (*n* = 21) had experienced more than 3. In terms of conception during sex work, 32.8% (*n* = 100) had conceived between 1 and 3 times, and 3.9% (*n* = 12) had conceived more than 3 times. Of those who had experienced unplanned pregnancies during sex work, 35.2% (*n* = 108) had experienced between 1 and 4 unplanned pregnancies, and 3.9% (*n* = 12) had experienced more than 4.

### Contraceptive use and methods mix

In total, 66.2% (95%CI 60.8–71.3) of the participants (208 out of 314) reported using dual contraception. Condom use in the last month was reported by 95.9% (*n* = 301 out of 314) of the participants, and 85.4% (*n* = 268) reported using a condom during their last sexual encounter. The frequency of condom use was reported as always by 21.7% (*n* = 68), sometimes by 76.4% (*n* = 240), and never by 1.9% (*n* = 6).

**Fig. 1:**
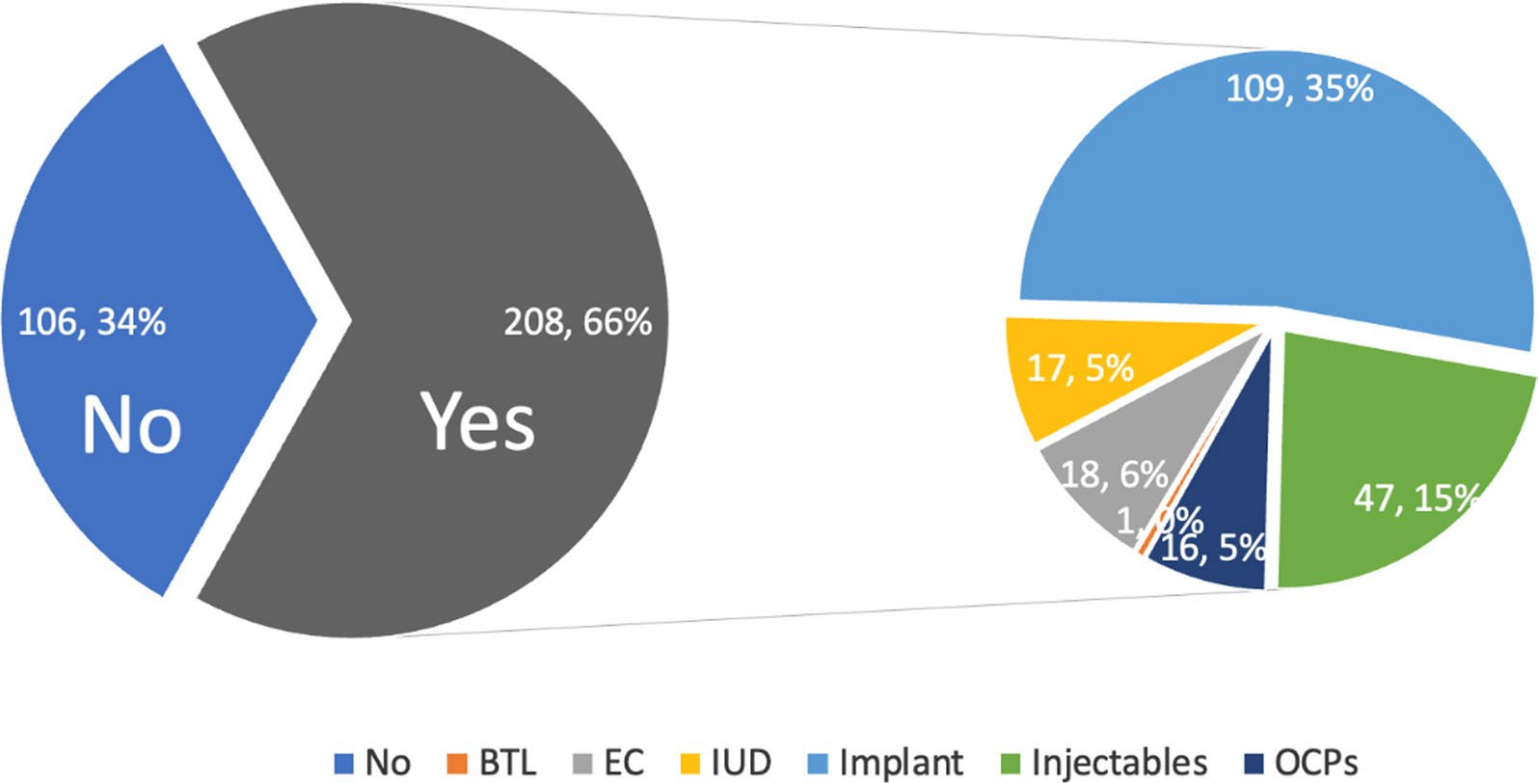
Pie-of-pie chart showing contraceptive methods used by female sex workers in Gulu city, Uganda. *BTL* bilateral tubal ligation, *EC* emergency pills, *OCPs* oral contraceptive pills

Among the participants, 208 reported using modern contraceptives. The most common method was the implants (52.4%, *n* = 109), followed by injectable contraceptives (22.6%, *n* = 47), and bilateral tubal ligation (0.5%, *n* = 1) was the least utilized, Fig. 1, Table 2.

**Table 2:** Contraceptive use among female sex workers in Gulu city, Uganda

Variable	Category	Frequency (*N* = 314)	Percent (%)
Dual contraception	No	106	33.8
	Yes	208	66.2
Condom use in the last month	No	13	4.1
	Yes	301	95.9
Condom use during last sexual encounter	No	46	14.6
	Yes	268	85.4
Frequency of condom use	Always	68	21.7
	Never	6	1.9
	Sometimes	240	76.4
Modern contraceptives *n* = 208	Bilateral tubal ligation	1	0.5
	Emergency pills	18	8.7
	Intrauterine device	17	8.2
	Implants	109	52.4
	Injectables	47	22.6
	Oral pills	16	7.7

### Factors associated with dual contraceptive use among female sex workers in Gulu city, Uganda

In the bivariable analysis, age (*p* < 0.001), ever had pregnancy (*p* < 0.001), ever had unplanned pregnancy (*p* = 0.032), and ever had unplanned pregnancy during sex work (*p* = 0.038) were significant.

In the multivariate analysis, ever had a pregnancy (adjusted PR: 1.87, 95%CI 1.40–2.51, *p* < 0.001) was significantly associated with dual contraceptive use. Participants who had ever had a pregnancy were 1.87 times likely to use dual contraception. Other demographic and reproductive factors, including education, residence, marital status, religion, ever had abortions, and history of abortion, were not significantly associated with the use of dual contraception (Table 3).

**Table 3:** Factors associated with dual contraceptive use among female sex workers in Gulu city, Uganda

Variable	Category	Dual Contraception		Crude PR	95% CI	*p* value
		Yes (*N* = 208)	No (*N* = 106)			
Age (years)	< 29	106 (57.9)	77 (42.1)	1		
	> 28	102 (77.9)	29 (22.1	1.34	1.15, 1.57	< 0.001
Education	None/Primary	105 (68.6)	48 (31.4)	1		
	Secondary/tertiary	103 (64.0)	58 (36.0)	0.92	0.80, 1.09	0.384
Residence	Urban	15 (71.4)	6 (28.6)	1		
	Rural	193 (65.9)	100 (34.1)	0.92	0.69, 1.22	0.575
Marital status	Married	34 (59.7)	23 (40.4)	1		
	Single	174 (67.7)	83 (32.3)	1.14	0.90, 1.43	0.28
Religion	Moslem	11 (47.8)	12 (52.2)	1		
	Christian	194 (67.8)	92 (32.2)	1.42	0.92, 2.19	0.115
Ever had a pregnancy	No	29 (39.7)	44 (60.3)	1		
	Yes	179 (74.3)	62 (25.7)	1.87	1.40, 2.51	< 0.001
Ever had an induced abortion	No	105 (62.9)	62 (37.1)	1		
	Yes	103 (70.1)	44 (29.9)	1.11	0.95, 1.30	0.178
Ever had a pregnancy during sex work, *n* = 305	No	124 (64.2)	69 (35.8)	1		
	Yes	82 (73.2)	30 (26.8)	1.34	0.98, 1.33	0.096
Unplanned pregnancies during sex work, *n* = 307	No	118 (63.1)	69 (36.9)	1		
	Yes	89 (74.2)	31 (25.8)	1.18	1.01, 1.37	0.038
Sex work as major source of income	No	120 (64.2)	67 (35.8)	1		
	Yes	88 (69.3)	39 (30.7)	1.08	0.92, 1.26	0.341
Depression	No	19 (61.3)	12 (38.7)	1		
	Yes	189 (66.8)	94 (33.2)	1.09	0.81, 1.46	0.565
HIV status *n* = 298	Negative	174 (67.7)	83 (32.3)	1		
	Positive	27 (65.9)	14 (34.2	0.97	0.77, 1.23	0.818
Alcohol consumption *n* = 310	Always	38 (73.1)	14 (26.9)	1		
	Never	4 (50.0)	4 (50.0)	0.68	0.34, 1.40	0.297
	Sometimes	162 (64.8)	88 (35.2)	0.89	0.73, 1.07	0.212
Age at first sexual encounter *n* = 286	< 17	106 (67.1)	52 (32.9)	1		
	> 16	87 (68.0)	41 (32.0)	1.01	0.86, 1.19	0.875

## Discussion

Dual contraception plays a pivotal role in the prevention of unintended pregnancies and STIs, including HIV among FSWs who are at heightened risk of these conditions due to the nature of their activities [18]. In this study, we aimed to determine the prevalence and associated factors with dual contraceptive use among FSWs in Gulu City, Northern Uganda. In this mainly urban setting in Northern Uganda, we found that almost 2 in 3 of the FSWs reported using dual contraception. In this study, the prevalence of HIV among FSWs was 13.8%, which is higher than in the general women population in Uganda at 6.3% [13]. Condom use in the last month was reported by 95.9%. However, the frequency of condom use was reported as always by only 22% and sometimes by majority (76.4%). The most commonly modern contraceptive method was the implants (51.5%) and injectable contraceptives (23.0%).

The prevalence of dual contractive use of about 67% in our study is comparable though slightly higher than findings from an earlier study conducted in the four regions of Uganda in 2017, where the prevalence of dual contraception among FSWs was estimated at 58.0% [3]**.** On the contrary, lower prevalence of dual contraceptives use were registered among FSWs in a more recent study in Northern Uganda (45%), Kenya (38%), Zambia (18.3%), Russia (12%), Switzerland (8%) and Tanzania (5.7–6%) [2, 9, 19–23]. The difference in the prevalence could be due to the joint efforts from TASO-Uganda and RHU in Gulu city, where evening mobile clinics are held to extend sexual and reproductive health services to FSWs to bridge the gap between FSWs and the health system. In addition, availability of condoms in the sex work places has been shown to increase use as FSWs can then negotiate for safe sex with clients [20].

Despite the ensured availability of condoms, most FSWs still report inconsistent use of condoms as was reported in other publications, where consistent condom use was as low as 16% [22]. This is attributed to factors such as being HIV positive, alcohol and substance use, clients’ indifference towards sex with condoms as well as mutual agreement between FSW and client to have condomless sex at an extra fee [24, 25]. As such, the risky behaviour increases odds of contracting HIV as evidenced by the high prevalence of HIV (13.8%) among FSWs in this study. Even more, FSWs who tested positive for HIV were less likely to use any condoms, propagating spread of HIV. As evidenced by the reduced uptake of contraceptive in FSW population with HIV in this study [22]. FSWs may be hard pressed to provide services in remote or unsafe occupational environments. The stigma and lack of protection place FSWs at increased risk of violence from clients, and because rushed negotiations, they are not able to screen clients properly or to negotiate the use of condoms in the sexual encounter [18, 26, 27]. Further studies are recommended to qualitative evaluate the barriers and facilitators of condom use among FSWs in Gulu City.

In our study, we found that over 70% of FSWs using modern contraceptives were using implants or injectable methods. Recent studies have shown that implants and injectables, which are long acting reversible contraceptives (LARCs), are the most preferred modern contraceptive methods especially for FSWs with future fertility desires due to their perceived effectiveness in prevention of unintended pregnancies and limited interference in the FSWs’ day-to-day activities [11, 28]. LARCs offer a reliable and convenient option for contraception, which is particularly important for FSWs who may face barriers to accessing healthcare services regularly.

We also observed that participants who had ever had a pregnancy were almost twofold more likely to use dual contraception, which is consistent with preliminary literature suggesting a similar association [2]. This finding suggests that pregnancy experience may play a role in shaping contraceptive decision-making among FSWs by increasing their awareness of the potential consequences of unprotected sex, such as the risk of unplanned pregnancies. Furthermore, the involvement of healthcare providers in providing education and counselling about the benefits of dual contraception during or after pregnancy may further influence their decision-making process regarding dual contraception [2].

In addition, women are more likely to use dual contraception if they are aware of dual contraception, have used non-barrier contraceptives as well as having disclosed their HIV status to their sexual partner given that they are HIV positive [29]. Whereas, having rush sexual negotiations owing to police presence and fear of side effects from modern contraceptive methods are negatively associated with dual contraceptive use [2].

Our study was not without limitations. Our study relied on self-reported contraceptive use and the information provided by the FSWs may have been influenced by recall bias and desire for social acceptance. Some studies have noted a trade off in women who adopt non-barrier modern contraception abandon condoms [30, 31]. However, our study provides further insights into contraceptive use among FSWs and sets a stage for further interventional studies.

## Conclusions

In this study, we found that about two-thirds of FSWs used dual contraception indicating a positive trend towards safer sexual practices. We found a positive association between previous pregnancy and dual contraception use, suggesting that FSWs who have experienced pregnancy prioritize the use of multiple contraceptive methods for enhanced protection against unplanned pregnancies and STIs.

### Recommendations

FSWs in Gulu city should be targeted for continued support for dual contraception among other sexual and reproductive health services through peer support networks and community-based interventions that address barriers to dual contraceptive use and promote its benefits. We also recommend development of targeted educational programs that emphasize and provide comprehensive information on dual protection, tailored to the specific needs of FSWs without history of pregnancy in Gulu city. Findings from this study should be utilized to guide the Ministry of Health in formulating evidence-based policies aimed at improving the uptake of dual contraception among FSWs to effectively prevent unintended pregnancies and improve the overall sexual and reproductive health outcomes for FSWs in Uganda.

## Data Availability

All relevant data are within the manuscript and its supporting information files. Data are available upon reasonable request from the first author.
